# Phenolic Compositions of Different Fractions from Coffee Silver Skin and Their Antioxidant Activities and Inhibition towards Carbohydrate-Digesting Enzymes

**DOI:** 10.3390/foods13193083

**Published:** 2024-09-27

**Authors:** Shiyu Dong, Lixin Ding, Xiuqing Zheng, Ou Wang, Shengbao Cai

**Affiliations:** 1 Faculty of Food Science and Engineering, Kunming University of Science and Technology, Yunnan Engineering Research Center for Fruit & Vegetable Products, Yunnan Key Laboratory of Plateau Food Advanced Manufacturing, Kunming 650500, China; 17865319331@163.com (S.D.); dlx2528@163.com (L.D.); 20202114018@stu.kust.edu.cn (X.Z.); 2 NHC Key Laboratory of Public Nutrition and Health, National Institute for Nutrition and Health, Chinese Center for Disease Control and Prevention, Beijing 100050, China

**Keywords:** coffee silver skin, phenolic compounds, antioxidant, α-glucosidase, α-amylase

## Abstract

Seeking food-derived antioxidants and inhibitors of α-glucosidase and α-amylase has been recognized as an effective way for managing diabetes. Coffee silver skin (CSS) is rich in phenolic compounds, which may be potential agents as antioxidants and for α-glucosidase and α-amylase inhibition. But whether phenolics in different forms show similar bioactivity remains unknown. In this study, phenolic compounds in CSS were extracted as free phenolics (FPs), esterified phenolics (EPs), and bound phenolics (BPs). The phenolic profiles and antioxidant activities of them were investigated. Their inhibitory effects on α-glucosidase and α-amylase were analyzed, and the inhibitory mechanisms were elucidated by molecular docking and molecular dynamic simulation. Results showed that FPs exhibited the best antioxidant ability and inhibitory effects on α-glucosidase and α-amylase. A total of 17 compounds were identified in FPs with 3-caffeoylquinic acid, 4-feruloylquinic acid, and dicaffeoylquinic acids as the dominant ones. Typical phenolics in FPs could bind to α-glucosidase and α-amylase through hydrogen bonds and form hydrophobic interaction with several key amino acid residues. In addition, 3,4-dicaffeoylquinic acid and 3-caffeoylquinic acid might be the principal components that account for the inhibitory effect of FPs on α-glucosidase. The results of this study may provide some scientific support for CSS utilization as a health-beneficial component in functional food development for type 2 diabetes mellitus management.

## 1. Introduction

According to the report of the International Diabetes Federation in 2021, over 530 million adults who aged 20–79 years old suffered from diabetes globally, and the prevalence was estimated to rise to 783 million by 2045 [1], which has become a huge health threat all over the world. As diagnosed by clinical standards, diabetes mellitus could be divided into type I diabetes mellitus and type II diabetes mellitus (T2DM). Among the patients with diabetes, over 90% of them are diagnosed as T2DM [2], and characterized by hyperglycemia, relative lack of insulin, or insulin resistance [3,4]. As one of the typical symptoms of T2DM, long-term hyperglycemia is associated with the elevated risk of many health issues, like diabetic retinopathy, peripheral neuropathy, renal damage, and even heart diseases [5,6]. Therefore, it is of great importance to control the blood glucose level for T2DM complications prevention.

As typical carbohydrate-digested enzymes, α-glucosidase and α-amylase participate in the hydrolysis of starch and sugars, and are closely related with glucose absorption and blood glucose level [7,8]. Therefore, inhibiting α-glucosidase and α-amylase activities are crucial in blood glucose level control. Clinical treatments, like acarbose or migilitol, are α-glucosidase and α-amylase inhibitors, but with un-ignored gastrointestinal adverse influence [9]. In cases like this, searching for safe and effective food-derived α-glucosidase and α-amylase inhibitors is worthy of more attention. In many published studies, plant-derived phenolic compounds have proved to be potential agents for inhibiting α-glucosidase and α-amylase activities [9,10]. 

In addition, antioxidant components are also beneficial for T2DM prevention or treatment. Prolonged hyperglycemia may impair the antioxidant defense system, induce oxidative stress, and, thus, worsen the situation of T2DM or its complications [11,12]. Due to their outstanding free radical scavenging capacity, phenolic compounds have been demonstrated to provide protective effects against T2DM and its associated complications by modulating various oxidative stress signaling pathways [13].

The coffee silver skin (CSS) is the outer layer of coffee seeds, and is usually treated as an industrial byproduct with large quantity. It was indicated that CSS preserves some nutritional properties of coffee [14], and is rich in polyphenols like chlorogenic acids [15]. Previous studies about CSS mainly focused on the extraction of different bioactive substances such as polyphenols, alkaloids, protein, and dietary fiber by various methods and evaluated their biological activities or functional characteristics [16,17]. The phenolic compositions, antioxidant capacity, and other bioactive properties of the CSS methanol or ethanol extracts have also been investigated to some extent by previous studies [18]. However, phenolic compounds in plants mainly occur in three different forms, namely, free phenols (FPs, mainly procyanidins and flavonoids), esterified phenols (EPs, mainly phenolic acids), and bound phenols (BPs, phenolics form covalent binding with plant cellulose, protein, or other macromolecules) [19]. Even in the same plant, the biological activities of polyphenols in different states would be different. For example, FP in barley has greater 2,2-Diphenyl-1-(2,4,6-trinitrophenyl) hydrazyl (DPPH) and 2′-Azinobis-(3-ethylbenzthiazoline-6-sulphonate (ABTS) free radical scavenging activity than BPs [20], the component BP of Water caltrop husk has the highest free radical scavenging ability, and FPs have the strongest glycosidase inhibitory activity [19]. These studies indicate that phenolics in different fovrms may show different contributions to different biological activities. Therefore, it is valuable to further clarify the composition and biological activity of polyphenols in different forms. 

Hence, in this study, phenolic compounds in different fractions of CSS are extracted as FPs, EPs, and BPs. The chemical compositions of the three fractions are identified, and the in vitro antioxidant activities are investigated. Meanwhile, their inhibitory effects on α-glucosidase and α-amylase are also evaluated, and the underlying inhibitory mechanisms of main phenolic compounds are further clarified through molecular docking and molecular dynamic simulation. The results of this study may provide some scientific proofs for the high-value utilization of CSS in the functional foods for T2DM management. 

## 2. Materials and Methods

### 2.1. Chemicals and Reagents

The CSS was obtained from Baoshan County, Yunnan Province (24°08′~25°51′ N, 98°05′~100°02′ E), and kept at 4 °C. During coffee processing, the green coffee bean was washed, peeled, dried, and the CSS was obtained. LC/MS grade methanol, acetonitrile, and formic acid were procured from Merck (Darmstadt, Germany). Folin–Ciocalteu reagent, ABTS, DPPH, and dinitro salicylic acid (DNS) were obtained from Macklin Biochemical Technology Ltd. (Shanghai, China). α-Glucosidase (≥50 units/mg protein) from *Saccharomyces cerevisiae* was procured from Shanghai Ryon Biological Technology Co., Ltd. (Shanghai, China). Porcine pancreatic α-amylase (>5.0 U/mg) was obtained from Shanghai Yuanye Biotechnology Company (Shanghai, China). Acarbose and p-nitrophenyl-α-D-glucopyranoside (p-NPG) were obtained from Beijing Solarbio Science & Technology Co., Ltd. (Beijing, China). Standards including (+)-catechin, (−)-epicatechin, rutin, etc., with purity more than 95% were purchased from Chengdu Must Bio-Technology Co., Ltd. (Chengdu, China). All other chemicals and reagents used in this study were of analytical grade.

### 2.2. Extraction of Different Phenolic Fractions

**Figure 1:**
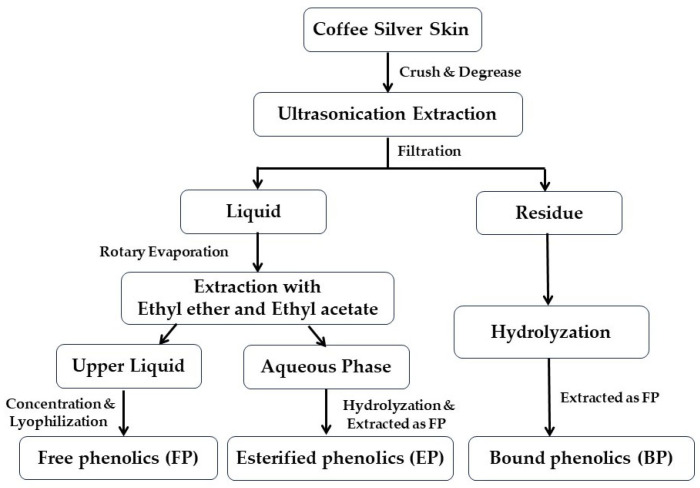
Workflow of CSS phenolic compounds extraction.

The CSS was pulverized and passed through a 40-mesh sieve, followed by extraction according to the previously described method [21] with slight modification (Figure 1). Briefly, 50 g CSS powder was degreased using petroleum ether three times, and then extracted with 250 mL solvent by ultrasonication for 30 min. The extract solvent was composed of 70% acetone (*v*/*v*) and 70% methanol (*v*/*v*) with a volume ratio of 1:1. After filtration, the residue was re-extracted twice, and the filtrate was concentrated with a rotary evaporator. Subsequently, the concentrated liquid was extracted with a 1:1 mixture of ethyl ether and ethyl acetate. The upper layer of the extraction liquid was collected, and the FP were obtained by concentration and lyophilization (Alpha 1-2 LD plus, Christ, Germany). The remaining aqueous phase was hydrolyzed with 4 mol/L sodium hydroxide for four hours at ambient temperature and the pH value was adjusted to 2.0 using hydrochloric acid. Then, the subsequent operation was the same as that of the FP to obtain the fraction of EPs. For the extraction of BPs, the filter residue was hydrolyzed with 4 mol/L NaOH for 4 h at room temperature and then filtered. The filtrate pH value was adjusted to 2.0, degreased with petroleum ether, and then treated with the same procedure as FPs. All extracts were stored at −20 °C.

### 2.3. Determination of Total Phenolic Content (TPC) and Total Flavonoid Content (TFC)

The TPC and TFC assays were conducted using a previous method [21]. For the TPC test, samples were completely dissolved in 80% methanol, and 1.0 mL of sample was added to the tube to thoroughly mix with 0.5 mL of Folin–Ciocalteu reagent. Then, 1.5 mL Na_2_CO_3_ (20% *m*/*v*) and 7.0 mL distilled water were added, and the tube was incubated in 70 °C water for 10 min. After cooling, the absorbance of the liquid was read at 765 nm with a SpectraMax M5 microplate reader (Molecular Device, Sunnyvale, CA, USA). The standard curve was prepared according to the absorbance value of gallic acid solution under the same conditions. The TPC of each sample was determined by reference to the calibration curve. For the TFC assay, 1.0 mL dissolved sample was successively mixed with ethanol (1.5 mL, 70%) and NaNO_2_ (0.15 mL, 5% *m*/*v*), and reacted for 5 min. Then, Al (NO_3_)_3_ (0.15 mL, 10% *m*/*v*) was added for a 6-min reaction, and followed by the addition of 1 mL NaOH and 0.20 mL ethanol. The reaction liquid was left ot stand for half an hour at room temperature, and its absorbance was read at 510 nm. The standard curve was drawn with rutin and used for the TFC quantification. 

### 2.4. Analysis of Antioxidant Activity

The DPPH and ABTS radical scavenging effects and ferric ion reducing antioxidant power (FRAP) of FPs, EPs, and BPs were determined based on our reported methods [22]. 

For the determination of DPPH free radical scavenging capacity, the sample was dissolved in 80% methanol (*v*/*v*) and diluted to five different concentrations. In each test tube, 2.0 mL DPPH (0.1 mol/L) and 0.5 mL test sample were mixed and incubated for 30 min in the dark. Then the absorbance of each sample was measured at 517 nm. 

To determine the ABTS radical scavenging ability, the sample was dissolved in 70% ethanol (*v*/*v*) and diluted to five different concentrations. A mixture was prepared by adding 0.5 mL test sample into 4.0 mL ABTS working solution, and then incubated at 30 °C for 6 min. The absorbance of the mixture was read at 734 nm. Ascorbic acid was employed as the positive control in the assay. 

For the FRAP assay, 4.5 mL of preheated FRAP working solution and 0.5 mL of sample were added to the test tube and incubated at 30 °C for 10 min. The absorbance of each sample was measured at 593 nm. This was plotted against the FeSO_4_·7H_2_O standard curve, and the FRAP value of each group was calculated.

### 2.5. Determination of Inhibitory Effect on α-Glucosidase and α-Amylase Activity

Basically, the inhibitory effects of FPs, EPs, and BPs on α-glucosidase activity were evaluated according to a published method [23]. In brief, a 50 mM sodium phosphate buffer (pH 6.8) was combined with 60 μL of α-glucosidase solution (0.5 U/mL) and 60 μL of sample solutions within a centrifuge tube, followed by incubation at 37 °C for 10 min. Then, 60 μL p-NPG solution (10 mM) was introduced to the reaction mixture in the tube and incubated at 37 °C for 30 min. Finally, the reaction was terminated by adding 180 μL Na_2_CO_3_ solution (0.2 mM), and the absorbance of the mixture was read by a SpectraMax M5 microplate reader at 405 nm.

For the α-amylase activity assay, sodium phosphate buffer (2 mM, pH 6.9) and 25 μL sample were mixed with 25 μL α-amylase solution (0.50 mg/mL) in the tube and incubated at 25 °C for 20 min. Then, 50 μL starch solution (1%) was introduced as substrate and the reaction system was incubated at 25 °C. After five min, 200 μL DNS reagent was added to the tube, followed by heating in boiling water for 10 min. When cooled down to room temperature, 200 μL solution was diluted with 1.0 mL distilled water, and its absorbance was measured at 540 nm. 

Each assay was performed in triplicate. Acarbose served as the positive control in the experiments. The enzyme inhibition rate was calculated as follows:

Enzyme activity inhibition (%) = [(A_control_ − A_sample_)/A_control_] × 100%

### 2.6. Characterization of Phenolics by UHPLC-ESI-HRMS/MS

The phenolic constituents in the three CSS fractions were characterized and quantified based on a previously reported approach [24]. The Thermo Fisher Ultimate 3000 UHPLC System (Thermo Fisher Scientific, Waltham, MA, USA) and Agilent Poroshell 120 SB-C18 column (2.7 μm, 2.1 mm × 100 mm, Santa Clara, CA, USA) were used for phenolic compounds separation. Mobile phase A consisted of 0.1% formic acid in water, while mobile phase B was acetonitrile. The mobile phase gradient elution procedure was as follows: 0–2 min, 5% B; 2–20 min, 5–50% B; 20–22 min, 50–70% B, and 22~25 min, 5% B. The flow rate of the mobile phase was 0.2 mL/min, the injection volume was 2 μL, and the temperature of the column was maintained at 30 °C. Mass spectrometric data were acquired in negative ionization mode with the following parameters: scanning range 100–1500 *m*/*z*, spray voltage 3.3 kV, capillary temperature 320 °C, and heater temperature 320 °C. The flow rates of auxiliary gas, sheath gas, and tail gas were 8.0 L/min, 32.0 L/min, and 4.0 L/min, respectively.

Phenolic compounds were identified by correlating mass spectral data, including *m*/*z* values, MS/MS fragment patterns, or retention times, with those of commercial standards or literature references. The identified compounds were then quantified or semiquantified using the external calibration curves established with the respective standards. 

### 2.7. Molecular Docking 

The molecular docking analysis was conducted to further identify the main α-glucosidase and α-amylase inhibitors among the three phenolic fractions of CSS. Since the 3D structure of α-glucosidase in *S. cerevisiae* was unavailable, the isomaltase of the same organism was used instead in the current work. The 3D structures of isomaltase (PDB ID: 3A4A) and α-amylase (PDB ID: 1OSE) were retrieved from the RCSB Protein Data Bank (http://www.rcsb.org/pdb/home/home.do, accessed on 9 February 2024), and the missing residues were complemented utilizing the Swiss-Spdbv software (Guex., 1996). The 3D structures of small ligands were sourced from the PubChem database (https://www.ncbi.nlm.nih.gov/pccompound, accessed on 9 February 2024), and structure optimization was conducted using the Avogadro program (Version 1.2.0) and the Generalized Amber Force Field (GAFF). Polar hydrogens and Gasteiger charges were assigned to both the small ligands and the α-glucosidase or α-amylase enzymes using the AutoDock Tools software (Version 1.5.7). Active binding sites were selected based on prior research findings [25,26].

### 2.8. Molecular Dynamics Simulation 

The GROMACS 19.5 package (https://manual.gromacs.org/, accessed on 10 February 2024) was used for molecular dynamics simulation, and the topological files of α-glucosidase and α-amylase were established using the Amber ff99SB-ILDN Force Field. In the molecular dynamics simulation, the system solvent was set in TIP3P water model and 0.15 M NaCl. Energy minimization was performed using the steepest descent algorithm, with the optimization criterion set to below 1000.0 kJ/mol/nm. The regular assembly simulation (NVT, 2 ns) was first performed, followed by isothermal and isobaric simulation (NPT, 1 ns), which ensured that the MD operation of the system was conducted at a constant temperature and pressure of 310.15 K and 1 bar, respectively. Then, 100 ns molecular dynamics was used for the simulation, which was accelerated by the GPU processor [27]. The data were extracted and the visualization image was processed by PyMol (Version 2.4). The stability of each compound combining with α-glucosidase or α-amylase was estimated by root mean square deviation (RMSD) and root mean square fluctuation (RMSF).

### 2.9. Statistical Analysis

Each experimental assay was conducted at least three times, and the data are presented as mean ± standard deviation (SD). The results were analyzed by one-way ANOVA followed by Tukey’s test (Origin Lab, Northampton, MA, USA). The statistical significance was set when the *p*-value was less than 0.05.

## 3. Results and Discussion

### 3.1. TPC and TFC of Different CSS Phenolic Fractions

Phenolic compounds are widely distributed in plants, but the forms are different. For example, in cashew nut testa and walnut skin, the phenolics are concentrated in free state [28,29], while in tea seeds, the majority of phenolic are in an insoluble bound form [30]. 

In this study, the TPC and TFC in CSS were assayed, and the results are shown in Table 1. In FPs, the TPC was 474.64 ± 22.76 mg gallic acid/g dry extract, and the TFC was 102.95 ± 8.13 mg rutin/g dry extract, which were both the highest among the three fractions (*p* < 0.05). As for EPs, the TPC in them was 1/4 that of FPs, while for TFC, it was one-half that of FPs (Table 1). In addition, BPs had the lowest TPC and TFC among the groups. The results of TPC and TFC indicate that the phenolic compounds in CSS were mainly concentrated in free form. 

Moreover, it was reported that the TPC in CSS ranged from 10 to 17 mg GAE/100 g [31], which was close to the result of EPs, but lower than that of FPs (Table 1). This difference was probably due to the diverse extraction and separation methods [32].

### 3.2. Antioxidant Capacity of Different CSS Phenolic Fractions 

The antioxidant potential of different CSS phenolic fractions was assessed by tests of ABTS radical scavenging ability, DPPH radical scavenging ability, and FRAP assays. As widely used free radical scavenging ability evaluation methods, the work mechanisms of DPPH and ABTS radical scavenging showed differences. Briefly, DPPH radical scavenging mainly reacted with organic free radicals, while ABTS mainly reacted with organic free radical cations, and their working pHs are 5–9 and 3–9, respectively [33]. Compared with DPPH assay, ABTS could react with a wider range of antioxidants, but showed poor stability [34]. Unlike these two methods, FRAP was a method for measuring antioxidant capacity based on electron transfer and mainly reacted with the Fe^3+^ complex. These three methods are simple and convenient, and have been widely used to determine the antioxidant capacity of polyphenols [33,34]. 

**Figure 2:**
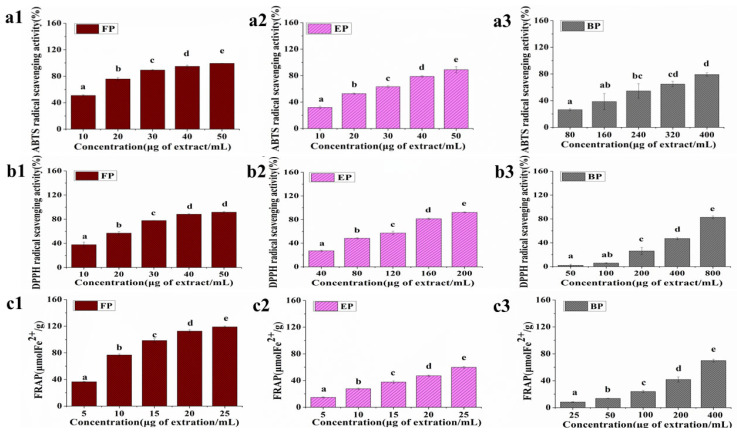
Antioxidant capacity of different CSS phenolic fractions. (**a1**–**a3**) ABTS radical scavenging activity; (**b1**–**b3**) DPPH radical scavenging activity; (**c1**–**c3**) FRAP analysis. Results are expressed as mean ± SD (n= 3). Mean values labeled with different letters denote significant differences (*p* < 0.05).

As per the results shown in Figure 2, FPs, EPs, and BPs exhibited antioxidative capacity in a dose-dependent manner in all the three tests. However, the antioxidant ability of the three fractions showed a marked difference. For example, at the concentration of 20 μg/mL, FPs could remove 75.82% of ABTS free radical, while EPs only achieved 52.82%, and BPs cleared less than 30% of ABTS free radical at the concentration of 80 μg/mL (Figure 2 (a1–a3)). Similarly, at a concentration of 10 μg/mL, the FRAP value of FPs was almost three times as high as EPs, and BPs showed an effect close to that of FPs only when the concentration reached 400 μg/mL (Figure 2(c1–c3)).

To accurately compare the antioxidant capacity between the three fractions, the IC_50_ value was calculated according to the results of ABTS assay and DPPH assay. In the ABTS assay, the IC_50_ values for FPs, EPs, and BPs were 14.98 ± 0.20 μg/mL, 23.58 ± 0.47 μg/mL, and 220.76 ± 4.65 μg/mL, respectively, and in the DPPH assay, the IC_50_ values for FPs, EPs, and BPs were 20.13 ± 0.59, 96.11 ± 3.26, and 448.05 ± 2.45 μg/mL, respectively. Therefore, it was obvious that the antioxidant capacities of the three fractions in CSS were in the order of FPs > EPs > BPs. It was reported that the antioxidant capacity was positively associated with the levels of TPC and TFC [35]. Hence higher TPC and TFC in FPs may account for their better antioxidant ability. Similarly, a previous study compared the antioxidant properties of coffee beans and their CSS collected in different countries; the higher TFC of CSS was also proven to be related to better antioxidant capacity [36].

### 3.3. Inhibitory Effects of CSS Phenolic Fractions on the Activities of α-Glucosidase and α-Amylase 

The α-glucosidase and α-amylase are the key enzymes in the digestion of carbohydrates and exert a significant influence in determining the level of glucose released [37]. Phenolic compounds demonstrate the ability to inhibit the activities of α-glucosidase and α-amylase, which makes them a potential agent for postprandial hyperglycemia control [38]. 

As shown by the data in Table 2, the IC_50_ value of α-glucosidase inhibition was 40.28 ± 1.40 μg/mL for FPs and 180.53 ± 4.23 μg/mL for EPs, which showed a significant difference (*p* < 0.05). As for the α-amylase inhibition, the IC_50_ value of FPs was 114.52 ± 3.62 μg/mL (Table 2). EPs showed weak inhibitory effects on α-amylase inhibition, and BPs showed no obvious suppression on both of the enzymes (Table 2). In addition, in FPs, the IC50 value of α-amylase inhibition was about 2.8 times that of α-glucosidase inhibition, which indicated the relatively stronger effect of FPs on α-glucosidase inhibition than that on α-amylase. 

It has been proven that the inhibition ability on α-glucosidase and α-amylase activity is positively associated with the level of phenolic compounds and the antioxidant activity of the plant extract [39,40]. Similarly, in this study, FPs showed the best digestive enzyme inhibitory effect, followed by EPs and BPs (Table 2). This order corresponded with the results of TPC and TFC assays and the antioxidant capacity (Table 1 and Figure 2).

Coffee was rich in polyphenols, and the whole coffee cherry extract inhibited the activities of α-glucosidase and α-amylase with the IC_50_ values of 1.71 mg/mL and 2.42 mg/mL, respectively [41]. Compared with this report, the IC_50_ values of FPs on α-glucosidase and α-amylase inhibition were much lower, indicating a potential better inhibitory effect. 

### 3.4. Identification and Quantitation of CSS Phenolic Compounds

**Figure 3:**
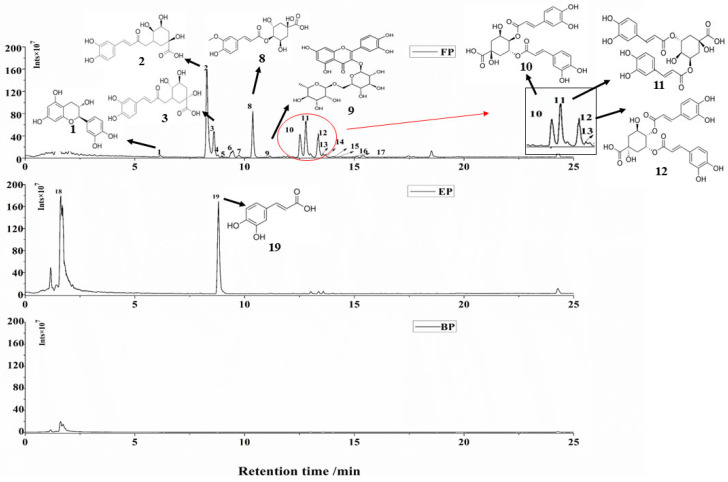
Peak chromatograms of three phenolics extracts. FP: free phenolics fraction; EP: esterified phenolics fraction; BP: bound phenolic compounds. Identification of the peaks and their mass spectrometry data are presented in Table 3.

Phenolic compounds present in plants with different forms and show different chemical compositions [42]. In this study, CSS phenolic compounds were extracted as FPs, EPs, and BPs, and the compositions of them were identified by UHPLC-ESI-HRMS/MS in negative ion mode. The chromatograms diagram is shown in Figure 3, and the mass spectrometry data are summarized in Table 3.

As shown in in Figure 3 and Table 3, a total of 17 compounds were identified in FPs, two compounds were detected in EPs, and no phenolic compound was found in BPs. Many researchers report that coffee bean is rich in chlorogenic acids [43], including various isomers and different conjugated structures [44]. As a part of the coffee bean, a consistent result of CSS analysis was obtained in this study. For FPs, compounds **2**, **8**, **10**, **11**, and **12** showed large peak areas (Figure 3), suggesting that these five phenolic compounds may be the dominant ones in FP extract. According to the mass spectrometry data in Table 3, compound **2** and compound **8** were characterized as 3-caffeoylquinic acid and 4-ferulicquinic acid, respectively, which are typical components of chlorogenic acids in coffee [44]. Compounds **10**, **11**, and **12** were identified as three isomers of dicaffeoylquinic acids, which also belong to coffee chlorogenic acids [15,45]. In addition, some chlorogenic acid derivatives were also found in FPs, like *p*-coumaroyl-caffeoylquinic acid and feruloyl-caffeoylquinic acid, but with low concentration (Table 3). As for the EPs, caffeic acid was detected to be the main phenolic compound (Figure 3 and Table 3), which was not reported in previous research. 

The phenolics identified in FPs and EPs of CSS were basically consistent with published reports, but the content was slightly different. Factors like varieties, agricultural practices, and processing methods may account for this difference [43].

### 3.5. Screening of Main α-Glucosidase and α-Amylase Inhibitors from FP 

As FPs exhibited the best inhibitory effects on digestive enzyme activities, the main identified compounds in them were applied to the molecule docking analysis. The absolute value of affinity indicated the potential strength of the small molecules binding to the enzymes. As the data shows in Table 4, 3,4-dicaffeoylquinic acids (3,4-diCQA), 3,5-dicaffeoylquinic acids (3,5-diCQA), rutin, 4,5-dicaffeoylquinic acid (4,5-diCQA), and 3-caffeoylquinic acid (3-CQA) showed high absolute value of affinity to *α*-glucosidase, and 3,5-diCQA, 4,5-diCQA, rutin, catechin, and epicatechin showed high absolute value of affinity to *α*-amylase, which represented good potential to bind with *α*-glucosidase or *α*-amylase and inhibited their activity. Therefore, these phenolic were applied for further molecule docking analysis. 

**Figure 4:**
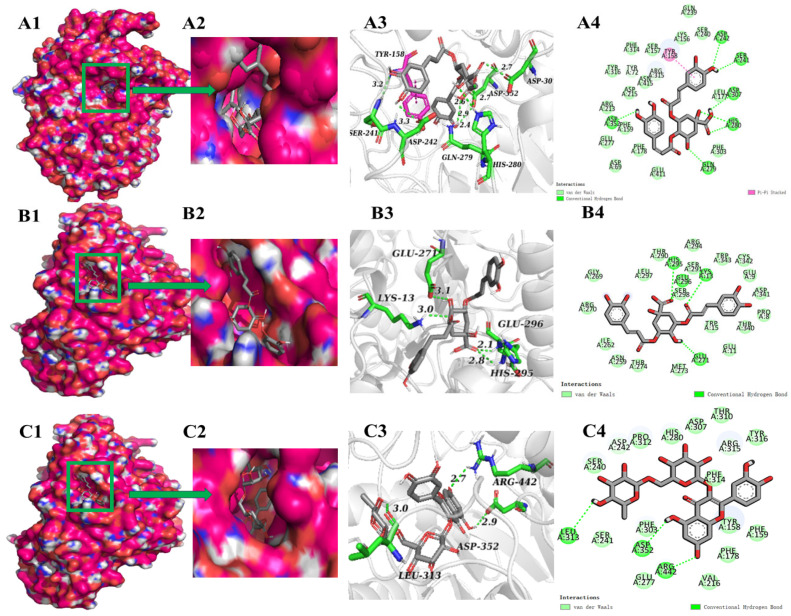

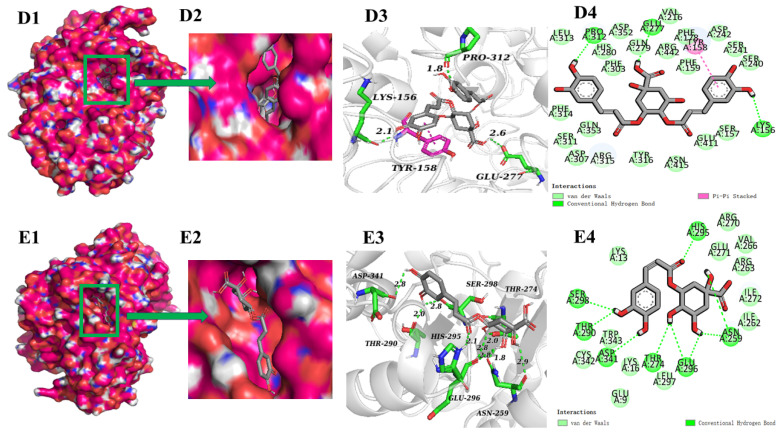
Molecular interactions of phenolic compounds with α-glucosidase. (**A**) 3,4-diCQA; (**B**) 3,5-diCQA; (**C**) rutin; (**D**) 4,5-diCQA; (**E**) 3-CQA. (**A1**–**E1**,**A2**–**E2**) Optimum docking conformations and binding site of phenolics with α-glucosidase; (**A3**–**E3**) 3D view of the interaction of phenolic compounds with the active binding site of α-glucosidase; (**A4**–**E4**) 2D view of the interaction of phenolic compounds with the active binding site of α-glucosidase.

**Figure 5:**
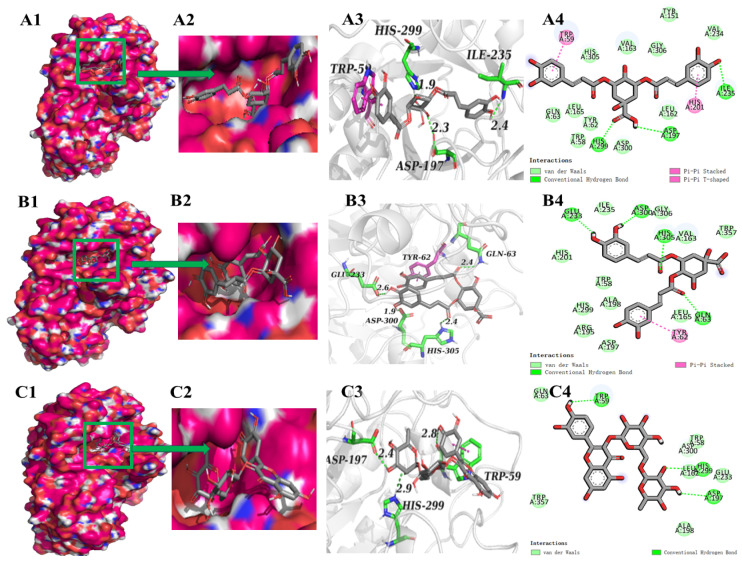

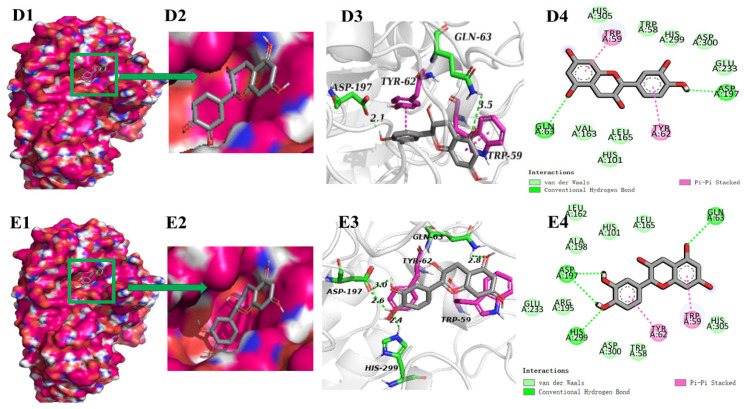
Molecular interactions of phenolic compounds with α-amylase. (**A**) 3,5-diCQA; (**B**) 4,5-diCQA; (**C**) rutin; (**D**) epicatechin; (**E**) catechin. (**A1**–**E1**,**A2**–**E2**) Optimum docking conformations and binding site of phenolics with α-amylase; (**A3**–**E3**) 3D view of the force of phenolic compounds on the binding site of α-amylase activity; (**A4**–**E4**) 2D view of the force of phenolic compounds on the binding site of α-amylase activity.

Molecular docking has been frequently used to investigate the intermolecular forces that mediate ligand–receptor interactions. In order to clarify the inhibitory mechanism, the binding sites and forces of the top five polyphenols in terms of absolute values of affinity to α-glucosidase or α-amylases were characterized (Figure 4 and Figure 5). Figure 4 shows the molecular docking results of phenolic compounds and α-glucosidase. During docking, these phenolic compounds were all encapsulated in the cavity of α-glucosidase, but it was not found that these phenolic compounds and α-glucosidase were bound in the same cavity (Figure 4(A1–E1)), which indicates that the mechanisms of the action may be different. In addition, the suppression of α-glucosidase activities by polyphenols is primarily due to the establishment of hydrogen bonds and hydrophobic interactions between these enzymes and the polyphenolic compounds [23]. The number of hydrogen bonds is pivotal in inhibiting the catalytic ability of the enzyme [46]. As shown in Figure 4, at the active site of α-glucosidase, 3,4-diCQA formed seven hydrogen bonds with amino acid residues Ser241, Asp242, Gln279, His280, Asp307, and Asp352 (Figure 4(A4)); 3,5-diCQA, rutin, and 4,5-diCQA formed three hydrogen bonds with amino acid residues (Figure 4(B4–D4)); and 3-CQA formed nine hydrogen bonds with amino acid residues Asn259, Thr274, Thr290, His295, Glu296, Ser298, and Asp341 (Figure 4(E4)). 3-CQA formed more hydrogen bonds but with relatively low binding energies, probably due to the fact that there were relatively few catalytically active sites for interaction with α-glucosidase. Among the five phenolic compounds, 3,4-diCQA and 3-CQA bound to the α-glucosidase active site with relatively more hydrogen bonds, indicating a strong potential to inhibit the α-glucosidase activity. As 3,4-diCQA and 3-CQA were identified to be the dominant phenolic compounds in FPs (Table 3), they might be the principal components that account for the inhibitory effect of FPs on α-glucosidase. In addition, in a previous molecule docking analysis, Asp242 was important in the catalytic site of α-glucosidase, and also an amino acid residue bind acarbose with α-glucosidase [47]. It was also found that Asp242 played an important role in this process in the experiment of Xing’s inhibitory activity on *Phyllanthus emblica* Linn. fruit and α-glucosidase [46]. In addition, Asp352 was also reported as a key active amino acid involved in α-glucosidase inhibition [48]. These two amino acids produced hydrogen bonds in the docking of α-glucosidase with 3,4-diCQA, which indicated that 3,4-diCQA had the ability to inhibit α-glucosidase activity, which was consistent with the published research [46,48,49]. Figure 5 exhibits the molecular docking results of the phenolic compounds with α-amylase complexes, indicating that the polyphenols could bind well into the cavities on the amylase surface (Figure 5(A1,A2–E1,E2)). At the pocket position of α-amylase, different polyphenols formed 2–4 hydrogen bonds with α-amylase, and the amino acid residues involved in the formation of hydrogen bonds involved Trp59, Gln63, Asp197, Glu233, Ile235, His299, Asp300, His305, etc. Furthermore, two aromatic residues, Trp59 and Tyr62, established π–π interactions with the polyphenol, which exhibited stacking characteristics at the active site’s portal. and occurred mainly between the amylopectin aliphatic and aromatic amino acids (including the indole ring) and the benzene ring of the polyphenols. It is noteworthy that the amino acid residues in the α-amylase active site, which were believed to be located at the position where the main interaction between the polyphenol inhibitor and the enzyme occurs, were also found in the present study, which may explain the α-amylase inhibitory activity of the phenolic compounds. Previous studies had shown that compounds interacting with α-amylase active residues Asp197, Glu233, and Asp300 may be active enzyme inhibitors [50]. These amino acid residues were also found in the results of the present molecular docking analysis (Figure 5), but in the same polyphenol–enzyme complex, these three active sites were not fully bound, and occurred more in the inactive site through hydrogen bonding and hydrophobic interactions, which may not be sufficient to exert an inhibitory effect [50]. This may account for the enhanced inhibitory effect of FPs on α-glucosidase compared to α-amylase.

### 3.6. Molecular Dynamic Simulation 

**Figure 6:**
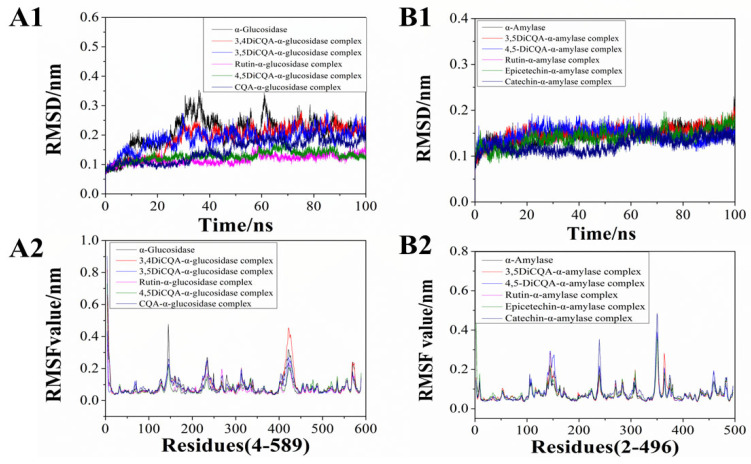
Molecular dynamics simulation results of complexes (100 ns). (**A1**,**B1**) Root mean square deviation (RMSD, 100 ns). (**A2**,**B2**) Root mean square fluctuation (RMSF, nm).

Molecular dynamic analysis could reveal the kinetics of binding interactions between small ligands and enzymes [51]. In the current study, the 100 ns molecular dynamic simulation was conducted to assess the stability of the complexes formed between phenolic compounds and α-glucosidase or α-amylase. RMSD was a parameter that measured the deviation between the initial structure and the simulated complex at the specific time; the results of it could reveal the stability of the complex [52]. A relatively lower RMSD value indicated a more stable combined system. The results in Figure 6(A1) show the RMSD value of the five phenolic compounds and the α-glucosidase combined system. It was found that the RMSD values of the five complexes increased in the initial 10 ns of the simulation. The 3,4-diCQA complex with α-glucosidase and 3.5-diCQA complex with α-glucosidase stabilized at 30 ns, with RMSD values around 0.22 nm. The rutin-α-glucosidase complex and the 4,5-diCQA-α-glucosidase complex reached stable conformation at 42 ns and 44 ns, respectively, and the complexes reached equilibrium with smaller RMSD values around 0.15 nm. The 3-CQA complex with α-glucosidase fluctuated more and reached stability later than the others complexed with the RMSD value of 0.18 nm. It was clear to see that the RMSD value of α-glucosidase demonstrated greater fluctuations compared to the five phenolics-bound complexes, suggesting that the phenolic compounds could form stable complexes with α-glucosidase and may further influence its activity. In addition, in Figure 6(B1), the variation of RMSD within 100 ns did not differ much between groups, indicating that phenolic compounds combined with α-amylase showed less influence on the stability. 

The fluctuation of RMSF described the flexibility of amino acid residues throughout the molecular dynamic simulation, and also revealed the effect of ligands on the protein structure [53]. The results in Figure 6(A2,B2) reflect the conformational flexibility of protein residues in relation to phenolics over time, as well as their mean deviation. The lower the RMSF value, the lower the flexibility of the amino acids, indicating the potential tighter structure of the protein–ligand complex. As for α-glucosidase (Figure 6(A2)), the overall RMSF fluctuation of the enzyme was slightly higher than those of the phenolics-α-glucosidase complexes, which means that the binding of the phenolic compounds may decrease the amino acids’ flexibility within the active site of the α-glucosidase and form a tight structure. A previous study indicated that there were three distinct structural domains of α-amylase: domain A encompasses residues 1–100 and 169–407, domain B includes residues 101–168, and domain C consists of residues 408–496 [54]. The results in Figure 6(B2) show that binding regions of phenolic compounds to α-amylase amino acid mainly focused on residue regions 120–160, 232–250, 340–360. Therefore, the phenolic compounds in the FP fraction of the CSS might mainly bind with the structure domain B of the α-amylase and influence its activity. Based on the above results, it could be seen that different polyphenols were able to bind more stably with glycosidase and amylase, and then inhibited the activities of the two enzymes, ultimately achieving the purpose of lowering blood glucose.

## 4. Conclusions

Among the three phenolic fractions of CSS, FPs showed the highest levels of TPC and TFC, and exhibited the best antioxidant ability and inhibition effects towards α-glucosidase and α-amylase. A total of 17 compounds were characterized in FPs, with 3-CQA, 4-feruloylquinic acid, and dicaffeoylquinic acids as the dominant ones. Findings from the molecular docking and molecular dynamics simulation indicated that typical phenolics in FPs could interact with α-glucosidase and α-amylase via hydrogen bonding and form hydrophobic interaction with several key amino acid residues. The major phenolic compounds formed a higher number of hydrogen bonds with α-glucosidase compared to α-amylase, which may illustrate the better inhibitory effect of FPs on α-glucosidase. 3,4-diCQA and 3-CQA might be the principal components that account for the inhibitory effect of FPs on α-glucosidase. The results of the present research may offer scientific rationale for the utilization of CSS as a health beneficial component in functional foods development for T2DM management. 

## Figures and Tables

**Table 1:** TPC and TFC levels in CSS phenolic fractions.

	TPC (mg GAE/g Dry Extract)	TFC (mg RE/g Dry Extract)
FP	474.64 ± 22.76 ^a^	102.95 ± 8.13 ^a^
EP	112.85 ± 8.38 ^b^	52.43 ± 1.21 ^b^
BP	33.96 ± 0.74 ^c^	14.83 ± 0.99 ^c^

Results are expressed as mean ± SD (n = 3). GAE means gallic acid equivalent; RE means rutin equivalent. Values denoted by different superscript letters within each column indicate significant difference among the samples (*p* < 0.05).

**Table 2:** Inhibition of CSS phenolic fractions on α-glucosidase and α-amylase activity.

	IC_50_ of α-Glucosidase Inhibition (μg/mL)	IC_50_ of α-Amylase Inhibition (μg/mL)
FP	40.28 ± 1.40 ^a^	114.52 ± 3.62
EP	180.53 ± 4.23 ^b^	>200
BP	>200	>200
Acarbose	0.63 ± 0.08	0.86 ± 0.07

Results are expressed as mean ± SD (n = 3). Values denoted by different superscript letters within each column indicate significant difference among the samples (*p* < 0.05).

**Table 3:** Identification of phenolic extracts of CSS by UHPLC-ESI-HRMS/MS.

Peaks	Compounds	TR (min)	[M-H]^−^ (*m*/*z*)	Molecular Formula	MS/MS Fragment Ions	Content (μg/g)	Extract
1	Catechin	7.91	289.0717	C_15_H_14_O_6_	123.0439, 109.0282	70.54 ± 2.89	FP
2	3-Caffeoylquinic acid	8.28	353.0875	C_16_H_18_O_9_	135.0440, 179.0345	24,163.04 ± 761.13	FP
3	5-Caffeoylquinic acid	8.65	353.0876	C_16_H_18_O_9_	191.0564, 151.0249	2663.01 ± 61.24	FP
4	Lactones of caffeoylquinic acid isomeride **1**	9.55	335.0772	C_16_H_16_O_8_	135.0440, 161.0234	23.56 ± 0.95	FP
5	Epicatechin	9.56	289.0717	C_15_H_14_O_6_	109.0282, 123.0438,	729.21 ± 23.27	FP
6	Para-coumaroyl-caffeoylquinic	9.70	337.0929	C_16_H_18_O_8_	173.0461, 63.0729	63.60 ± 1.83	FP
7	Lactones of caffeoylquinic acid isomeride **2**	9.90	335.0773	C_16_H_16_O_8_	135.0439, 161.0233	32.98 ± 0.65	FP
8	4-Feruloylquinic acid	10.37	367.1021	C_17_H_20_O_9_	173.0459, 134.0283	12,529.73 ± 325.77	FP
9	Rutin	11.39	609.1470	C_27_H_30_O_16_	300.0273, 301.0345	39.25 ± 0.423	FP
10	3,4-Dicaffeoylquinic acid	12.53	515.1176	C_25_H_24_O_12_	179.0274, 173.0450	6515.49 ± 134.21	FP
11	3,5-Dicaffeoylquinic acid	12.73	515.1173	C_25_H_24_O_12_	191.0915, 179.0353	9822.35 ± 214.13	FP
12	4,5-Dicaffeoylquinic acid	13.36	515.1176	C_25_H_24_O_12_	173.0777, 179.0352,	6076.10 ± 123.89	FP
13	3-*p*-Coumaroyl-caffeoylquinic acid	13.83	337.0928	C_16_H_18_O_8_	119.0489, 163.0390	159.23 ± 3.80	FP
14	Feruloyl-caffeoylquinic acid isomeride **1**	13.91	529.1351	C_26_H_26_O_12_	173.0445, 193.0500	257.28 ± 7.87	FP
15	Feruloyl-caffeoylquinic acid isomeride **2**	14.06	529.1351	C_26_H_26_O_12_	173.0445, 193.0498	497.70 ± 10.44	FP
16	Feruloyl-caffeoylquinic acid isomeride **3**	14.37	529.1351	C_26_H_26_O_12_	191.0552, 179.0340	573.81 ± 6.13	FP
17	Feruloyl-caffeoylquinic acid isomeride **4**	14.85	529.1351	C_26_H_26_O_12_	173.0445, 191.0552	428.87 ± 12.92	FP
18	Unknown	0.83	-	-	-	-	EP
19	Caffeic acid	7.69	179.0034	C_9_H_8_O	135.0021, 134.1002	16,325.27 ± 359.16	EP

TR: retention time; FP: only detected in free phenolic extracts; EP: only detected in esterified phenolic extracts. (+)-Catechin standard was used for the quantification of compounds **1**, **4**, **7**. 3-Caffeoylquinic acid standard was used for the quantification of compound **2**. 5-Caffeoylquinic acid standard was used for the quantification of compound **3**. (−)-Epicatechin standard was used for the quantification of compound **5**. *P*-coumaric acid standard was used for the quantification of compounds **6**, **13**. Ferulic acid standard was used for the quantification of compounds **8**, **14**–**17**. Rutin standard was used for the quantification of compound **9**. 3,4-dicaffeoylquinic acid standard was used for the quantification of compound **10**. 3,5-dicaffeoylquinic acid standard was used for the quantification of compound **11**. 4,5-dicaffeoylquinic acid standard was used for the quantification of compound **12**. Caffeic acid standard was used for the quantification of compound **19**.

**Table 4:** Results of molecular docking.

Peaks	Pubchem ID	Phenolics	Affinity of α-Glucosidase (kcal/mol)	Affinity of α-Amylase (kcal/mol)
1	9064	Catechin	−8.5	−9.0
2	1794427	3-Caffeoylquinic acid	−9.5	−8.1
3	12310830	5-Ccaffeoylquinic acid	−9.1	−8.2
4	73160	Epicatechin	−8.5	−9.0
5	5281766	Para-coumaroyl-caffeoylquinic	−8.9	−8.1
6	10177048	4-Feruloylquinic acid	−8.4	−8.1
7	5280805	Rutin	−10.3	−9.1
8	5281780	3,4-Dicaffeoylquinic acid	−10.6	−8.5
9	6274310	3,5-Dicaffeoylquinic acid	−10.5	−9.1
10	6474309	4,5-Dicaffeoylquinic acid	−10.1	−9.1
11	9945785	3-*p*-Coumaroyl-caffeoylquinic acid	−8.5	−8.4

## Data Availability

The original contributions presented in the study are included in the article, further inquiries can be directed to the corresponding authors.
